# *Eleutherine plicata* Herb. and Its Promising Constituents Amoebicides

**DOI:** 10.1021/acsomega.5c03735

**Published:** 2025-09-16

**Authors:** Ana Laura Gadelha Castro, Mírian Letícia Carmo Bastos, Gleison Gonçalves Ferreira, Renilson Castro de Barros, Renato Araujo da Costa, José Eduardo Gomes Arruda, Maria Fâni Dolabela

**Affiliations:** 1 Postgraduate Program in Pharmaceutical Innovation, Institute of Health Sciences, 37871 Federal University of Pará, Belém, Pará 66075-110, Brazil; 2 Postgraduate Program in Biodiversity and Biotechnology, 37871 Federal University of Pará, Belém, Pará 66075-110, Brazil; 3 Postgraduate Program in Pharmaceutical Sciences, Institute of Health Sciences, 37871 Federal University of Pará, Belém, Pará 66075-110, Brazil; 4 Federal Institute of Education Sciences of the State of Pará, Campus Abaetetuba, Abaetetuba, Pará 68440-000, Brazil; 5 Institute of Health Sciences, 37871 Federal University of Pará, Belém, Pará 66075-110, Brazil

## Abstract

*Eleutherine plicata* is used in the Amazon region for the treatment of amoebiasis. The plant has been found to contain naphthoquinones (eleutherin and isoeleutherin) and naphthalene derivatives (eleutherol and isoeleutherol), which have been isolated. This study investigated, through molecular docking, the potential of isoeleutherin, eleutherin, eleutherol, and isoeleutherol against different proteins of *Entamoeba histolytica*. The potential amoebicidal effects were assessed by examining the interaction with two proteins, O-acetyl-serine sulfhydrylase (3BM5) and thioredoxin reductase (4CCQ), obtained from the public RCSB PDB database. The proteins were optimized using the APBS server, which added charges, polar hydrogens, water molecules, and cocrystallized components. The enzymes were subsequently removed. In this study, the amoebicidal potential of the molecules isoeleutherin, eleutherin, isoeleutherol, and eleutherol was evaluated. In *in silico* studies, in MM/GBSA energy calculations, isoeleuterin was the most promising ligand (−27.67 kcal/mol) in the EhTrxR target, while in the EhOASS target, it maintained a competitive binding energy (−20.30 kcal/mol), being the most promising natural compound. In addition, it has a lower toxicity profile. The search for therapeutic alternatives for the treatment of amoebiasis is important, with *in silico* studies being one strategy. Isoeleuterin is the most promising molecule for *in vitro* and *in vivo* validation studies.

## Graphical abstract

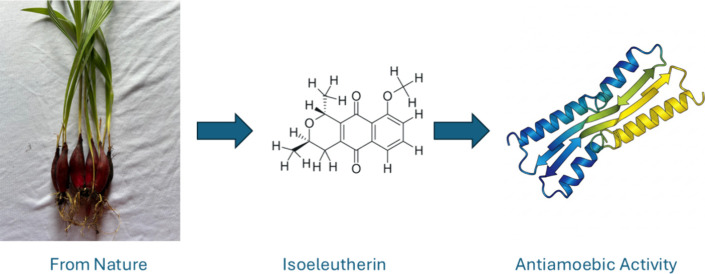

## 1 Introduction

Amebiasis is typically caused by the ingestion of food or water contaminated with *Entamoeba histolytica* cysts.^1^ *Entamoeba histolytica* is a single-celled human parasite that moves through ′pseudopodium′. While the infection may not always present symptoms, it can lead to severe intestinal and/or life-threatening extraintestinal disease.^2^ Following excystation, the formed trophozoites migrate to the large intestine, where they have the potential to become virulent and invasive. This can result in the breakdown of the mucosal epithelial barrier, inducing overproduction of mucus, causing inflammation, and destruction of host cells, ultimately leading to amoebic colitis.^1^

Amebiasis is a disease that affects approximately 500 million people annually, with about 10% presenting the pathogenic form of the disease, resulting in approximately 100,000 deaths.^3^ This disease is endemic in developing countries in Central and South America, Africa, and Asia, with the most affected countries being Bangladesh, India, Brazil, Colombia, Mexico, and China are the most affected countries.^4^ However, the true prevalence of amoebiasis in these countries is currently unknown due to the scarcity of studies and often limited diagnostic and surveillance capacity in areas where *E. histolytica* is endemic.^5^

The parasite exhibits a complex life cycle with antigenically diverse stages that enable it to evade the host’s immune system. In addition, it possesses several factors that contribute to its virulence, such as resistance to complement, the ability to eliminate ROS and NOS, and a capacity for oxygen reduction.^6^

The objective of the treatment is to eliminate parasite invasion and prevent the disease from becoming invasive by stopping its intestinal transport. Currently, there are several drugs available for treatment, including teclozam and nitroimidazoles secnidazole, metronidazole, and tinidazole. Teclozam is recommended for asymptomatic patients or those with mild forms of the disease,^7^ while secnidazole is used to treat intestinal forms of the disease. However, it is important to note that secnidazole should be avoided during the first trimester of pregnancy and while breastfeeding. Metronidazole is a viable option for treating severe intestinal forms of the disease. It is important to be prudent when using metronidazole, as gastrointestinal, psychiatric, neurological, and visual disturbances have been reported, including extraocular muscle paresis and diplopia, have been reported with its use.^8,9^

Tinidazole is considered as a viable alternative treatment for extraintestinal forms, with a spectrum and frequency of side effects similar to those of metronidazole 7. Additionally, resistance to nitroimidazoles is becoming more common due to the presence of resistance genes (nim A to nim J).^10^

To address these issues, it is crucial to explore therapeutic alternatives. Medicinal plants are a valuable source of new drugs. An ethnobotanical study showed that *E. plicata* Herb tea is utilized by Amazonian communities to treat diarrhea and amoebiasis.^11^ Chemical studies carried out on the ethanolic extract obtained from *E. plicata* bulbs led to the isolation of the isoeleutherin, eleutherin, eleutherol^12,13^ and isoeleutherol^14^ eleutherinone (R) – 4-Hydroxyeleutherine; eleutherone; isoeleuthoside C; eleutherinol-8-O-β-d-glucoside^14,15^ (Figure 1).

**Figure 1:**
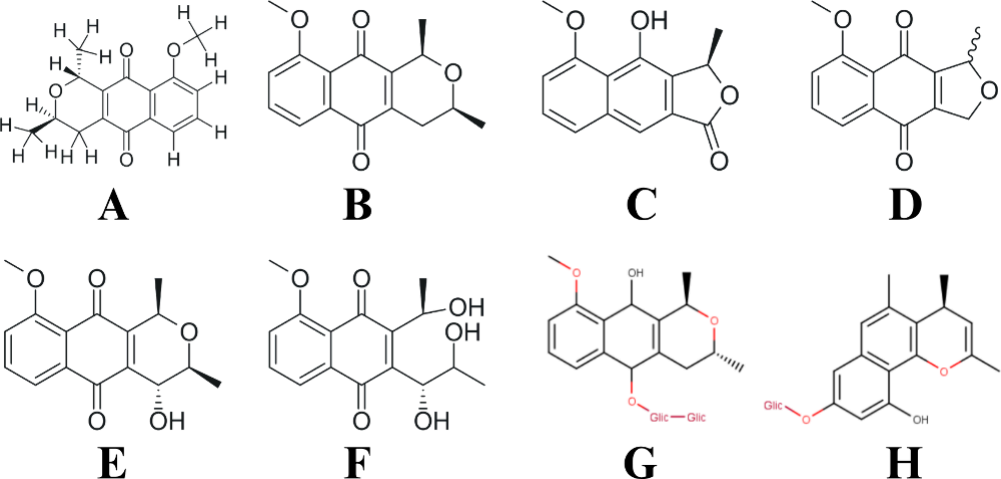
Chemical constituents isolated from *Eleutherine plicata*: (A) isoeleutherin; (B) eleutherin; (C) eleutherol; (D) eleutherinone; (E) (R) – 4-Hydroxyeleutherine; (F) eleutherone; (G) isoeleuthoside C; and (H) eleutherinol-8-O-β-d-glucoside.

The literature shows that these compounds have promising antiprotozoal activity, especially about antileishmanial activity^16^ and antimalarial^17^ Isoeleutherin and eleutherin demonstrated antiplasmodial activity in an *in vitro study* ^13^ Eleutherol showed more promise than the naphthoquinones isoeleutherin and eleutherin in mice infected with *Plasmodium berghei*

Previous studies have evaluated the binding of isoeleutherin and other compounds to different proteins through molecular docking. The molecular docking of eleutherin and isoeleutherin showed antiplasmodial activity with a mechanism similar to that of atovaquone. They were able to interact with the cytochrome bc1 complex.^13,17^

Isoeleutherin and its analogue were evaluated for their leishmanicidal potential by docking with molecular Trypanothione Redutase. The results showed stability and favorable interactions with TR, indicating a promising compound for leishmanicidal activity.^17^

Naphthoquinones have long been associated with oxidative stress, and this potential was evaluated by using molecular docking with various targets, including eleutherol. The results showed that eleutherin and isoeleutherin had the lowest binding energy for catalase (CAT), glutathione reductase (GR), and glutathione peroxidase (GPx1) among the compounds tested.^16^

The in vitro activity of isoeleutherin against *Entamoeba hystolitica* and *Entamoeba dispar*, indicating its potential role in combating amebiasis.^18^ As it is a quinolic compound, amebicide activities may be related to oxidative stress, i.e., involving the formation of reactive oxygen species (ROS) such as hydrogen peroxide (H_2_O_2_), superoxide radical anion (O_2_^–.^), and hydroxyl radical (HO·) induced by the bioreduction of the quinonic complex or the induction of apoptosis. As a consequence, cells lose their ability to repair, determining the potential of these compounds to cause DNA damage by breaking single and/or double strands and studies have shown mechanisms of intercalation in the double helix of DNA and alkylation of the nucleotides.^19^

The potential of isoeleutherin, eleutherin, and eleutherol for amoebae has yet to be evaluated by molecular docking. Designing new drugs requires the identification and functional characterization of molecular targets for amoebae, making this research highly relevant. *E. histolytica* O-acetyl-serine sulfhydrylase (EhOASS) and thioredoxin reductase (EhTrR) represent two well-established molecular targets.^20^ The processes involved in redox metabolism are of particular interest in amoebiasis.^21^

The EhOASS catalyzes the final step of the cysteine biosynthetic pathway, which is essential for detoxifying the effects of ROS and oxygen, as well as for trophozoite fixation and growth^6^ EhOASS catalyzes the final step of the cysteine biosynthetic pathway, which is essential for detoxifying the effects of ROS and oxygen, as well as for trophozoite fixation and growth.^20^ Thioredoxin reductase is an important target for new drugs because it plays a role in parasite survival via the parasite’s anaerobic cysteine (thiol) system. To do this, it reduces NADPH via FAD, where the NADPH molecule rotates in the active site, changing the conformation of the protein, allowing it to donate electrons to the thioredoxin system or even promote the alteration of active disulfide bridges via FADH2.^22^ Activation of this protein increases the susceptibility of *E. hystolitica* trophozoites to death caused by oxidative stress.^22^ This supports the hypothesis that naphthoquinones’ amoebicidal activity involves this mechanism.^22^

This study utilizes molecular docking simulation to investigate the binding interactions between isoeleutherin, eleutherin, eleutherol, and isoeleutherol with a specific target of *E. histolytica*. The objective is to evaluate the amoebicidal potential of these molecules.

## 2 Materials and Methods

### 2.1 Choice of Molecules and Target

The antiparasitic activity of *E. plicata* has been associated with the naphthoquinones isoeleutherin and eleutherin.^17^ However, an *in vivo* study in mice infected with *Plasmodium berghei* demonstrated the potential of eleutherol.^16^ Isoeleutherol is considered one of the chemical markers of *E. plicata* ^14^ but there is a lack of studies evaluating the antiparasitic activity of this compound. In light of this, the following molecules were selected: eleutherin, eleutherol, isoeleutherin, and isoeleutherol.

A previous study demonstrated the involvement of oxidative stress in the antileishmanial activity of *E. plicata* ^17^ Therefore, enzymes present in *E. histolytica* involved in the detoxification of ROS were selected: O-acetyl-serine sulfhydrylase (EhOASS) and thioredoxin reductase (EhTrR).

### 2.2 Docking Molecular

The chemical structures of the compounds eleutherin, eleutherol, isoeleutherin, and isoeleutherol and the reference drug metronidazole were obtained from the PubChem database and optimized using the DFT/B3LYP/cc-pVDZ quantum method with the Gaussian09 software. The proteins O-acetyl-serine sulfhydrylase (3BM5, resolution of 2.40 Å) and thioredoxin reductase (4CCQ, resolution of 1.50 Å) were downloaded from the public RCSB PDB database (https://www.rcsb.org).

Molecular docking was performed using Molegro Virtual Docker (MVD) software, version 5.5. The coordinates for the spheres were set at x = 13.57, y = 38.36, and z = −4.71 for O-acetyl-serine sulfhydrylase (3BM5) and at x = −7.69, y = −9.92, and z = −12.37 for thioredoxin reductase (4CCQ), with a radius of 15 Å. The interaction evaluation was performed using the MolDock evaluation function.

Since the proteins do not have cocrystallized agonists or antagonists, cofactors were used for the redocking. The O-acetyl-serine sulfhydrylase (3BM5) was validated using PLP with an RMSD of 1.28 Å, and thioredoxin reductase (4CCQ) using ODE with an RMSD of 0.8 Å, validating the docking protocol.

The metronidazole, a well-known amoebicidal agent, allowed for comparative docking analysis with the natural compounds, providing a reference for binding energy, interaction profiles, and active site engagement.

### 2.3 Dynamic Molecular

To analyze the conformational changes and stability of the ligand-protein complexes, Molecular Dynamics (MD) simulations were performed for the unbound form of the O-acetyl-serine sulfhydrylase (3BM5) and thioredoxin reductase (4CCQ) proteins as well as for the complexes of these proteins with the compounds eleutherin, eleutherol, isoeleutherin, isoeleutherol, and the reference drug metronidazole.

The AMBER24 simulation package was used to perform 200 ns MD simulations on all complexes, using the GPU-accelerated version of the Partial Mesh Ewald Molecular Dynamics (PMEMD) module.^23^ The atomic charges of the ligands were calculated using the restricted electrostatic potential (RESP) protocol, at the theoretical level of HF/6–31G*, using Gaussian 09 software. The PDB 2PQR server^24^ was used to determine the protonation state of the amino acid residues at pH 7.4. In the preparation of all systems, the proteins and ligands were represented by the ff19SB^25^ and GAFF^26^ force fields, respectively. All systems were solvated in the tLeap module using an octahedral water box with the TIP3P model,^27^ and counterions were introduced to neutralize the system charges and achieve a physiological concentration of 0.15 M. This procedure was adopted to replicate physiological conditions, ensuring a more suitable ionic strength for obtaining biologically relevant results.

Each system was minimized in four stages, with 5000 steepest descent minimization steps followed by 5000 conjugate gradient steps. In the first stage, only the ions and water molecules were minimized; in the second stage, the hydrogen atoms of the protein structures; in the third, both the hydrogen atoms and the water molecules; and finally, all atoms of the systems.

The systems were heated from 0 to 300 K by performing 200 ps of MD and then 300 ps of density equilibration with positional restraints on the protein–ligand atoms in constant volume. All systems were equilibrated with 1 ns of MD without positional restraints at a constant pressure. The temperature was maintained at 300 K by coupling to a Langevin thermostat using a collision frequency of 2 cm^–1^. The 10 Å cutoff for the systems was used for nonbonded interactions, and the Particle Mesh Ewald (PME) method^28^ and the SHAKE algorithm^29^ were used to restrain bond lengths involving hydrogen atoms. Finally, the production MD simulations were performed using 200 ns at a temperature of 300 K without positional restraints. The generated trajectories were used to analyze the behavior of each complex and were employed for calculating the free binding energy.

#### 2.3.1 Binding Free Energy Calculation Using MM/GBSA

To clarify the binding affinity of each simulated ligand with the protein, binding free energy calculations were performed using the MM-GBSA method.^30^ The last 10 ns of the MD simulation trajectories were used to calculate the binding free energy, employing the AmberTools24 package.^31,32^

## 3 Results

### 3.1 Molecular Docking Results

To investigate the inhibitory potential of the compounds eleutherin, eleutherol, isoeleutherin, and isoeleutherol against the proteins O-acetyl-serine sulfhydrylase (EhOASS) and thioredoxin reductase (EhTrR) of *Entamoeba histolytica*, molecular docking studies were conducted. The results demonstrated favorable interactions between the compounds and key residues of the active sites of both target proteins, suggesting their potential as viable inhibitors.

Figure 2 illustrates the binding interactions of the compounds with the EhTrR protein. The reference drug metronidazole formed hydrogen bonds with residues Ser12, Gly13, Glu34, Ala38, Gly44, and Ala119, as well as π-alkyl interactions with Val41 and alkyl interactions with Ala41. These interactions highlight its known efficacy against *E. histolytica* and serve as a benchmark for comparison with those of the tested natural compounds.

**Figure 2:**
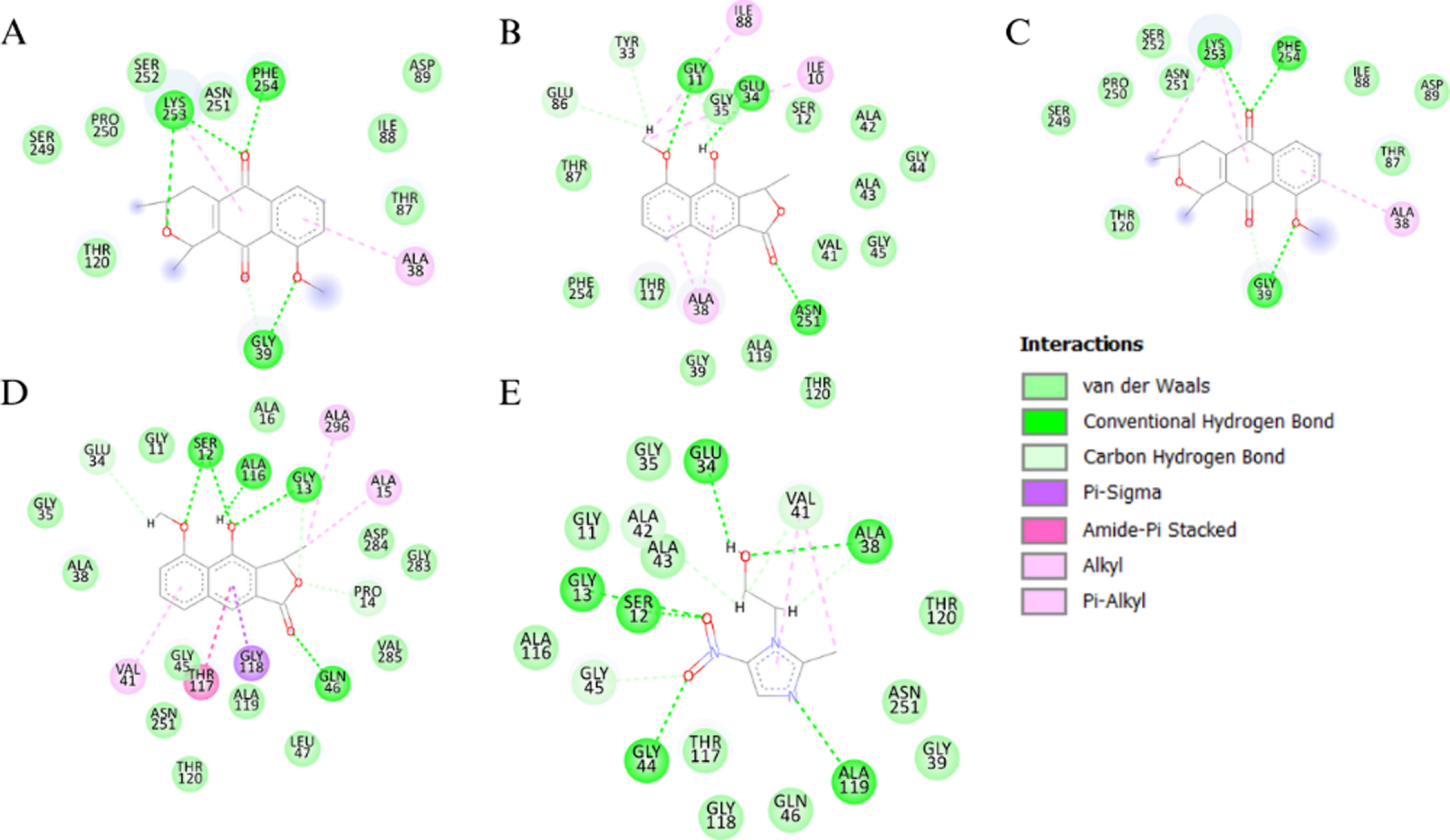
Interactions between Eleutherin (A), Eleutherol (B), Isoeleutherin (C), Isoeleutherol (D) and Metronidazole (E) with the enzyme thioredoxin reductase (4CCQ). Once they are analogous, the molecules bind to the same amino acid residues.

Among the natural products, eleutherin exhibited hydrogen bonds with Gly39, Lys253, and Phe254, and π-alkyl interactions with Ala38 and Lys253. Eleutherol formed hydrogen bonds with Gly11, Glu34, and Asn251, in addition to alkyl interactions with Ile88 and Ile10 and π-alkyl interactions with Ala38. Isoeleutherin interacted through hydrogen bonds with Lys253, Phe254, and Gly39 and showed π-alkyl interactions with Ala38. Isoeleutherol established hydrogen bonds with Ser12, Ala116, Gly13, and Gln46, as well as π-alkyl interactions with Ala41 and alkyl interactions with Ala296 and Ala15.

To explore the inhibitory potential of the selected compounds on the enzyme O-acetyl-serine sulfhydrylase (EhOASS), additional molecular docking analyses were performed. The results revealed significant interactions between the natural molecules and key residues of the enzyme’s active site, suggesting a potential modulatory effect on its catalytic activity.

Figure 3 depicts the binding interactions of the compounds with the EhOASS protein. The reference drug metronidazole formed hydrogen bonds with residues Ser189, Thr193, Ser194, and Thr196 as well as π-alkyl interactions with Pro307 and alkyl interactions with Ile305. These interactions, underscore its established efficacy against *E. histolytica* and serve as a comparative baseline for assessing the binding behavior of the tested natural compounds.

**Figure 3:**
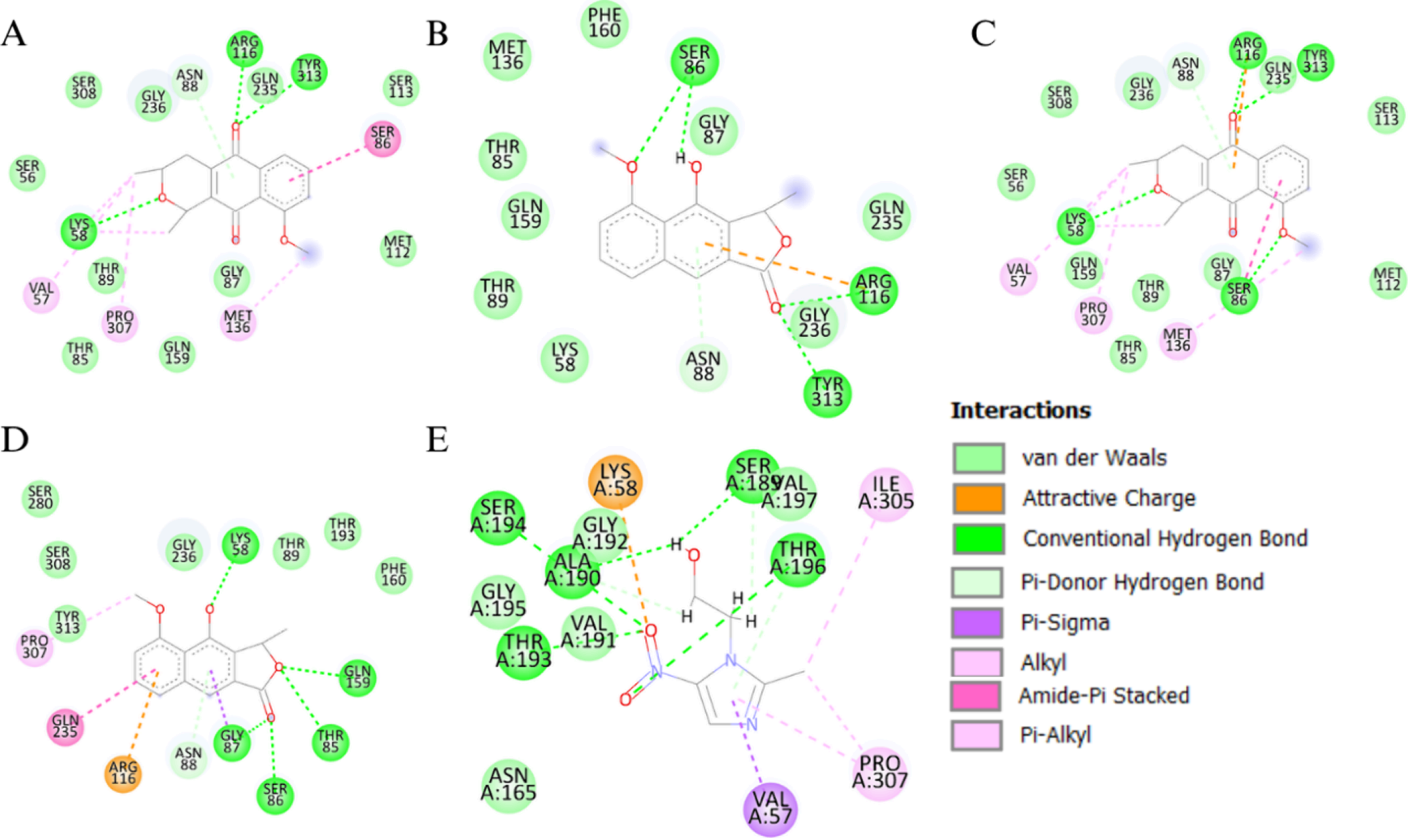
Interactions between Eleutherin (A), Eleuterol (B), Isoeleutherin (C), Isoeleutherol (D) and Metronidazole (E) with the enzyme O-acetyl-serine sulfhydrylase (3BM5). Once they are analogous, the molecules bind to the same amino acid residues.

Among the natural products, eleutherin established hydrogen bonds with Arg116, Tyr313, and Lys58, along with π-alkyl interactions with Ser86 and alkyl interactions with Met136, Pro307, and Val57. Eleutherol formed hydrogen bonds with Ser86, Arg116, and Tyr313, engaging residues similar to those targeted by eleutherin. Isoeleutherin exhibited hydrogen bonds with Arg116, Tyr313, Lys58, and Ser86, in addition to alkyl interactions with Val57, Pro307, and Met136—highlighting a broad interaction profile within the active and adjacent regulatory regions of the enzyme. Isoeleutherol, in turn, interacted through hydrogen bonds with Lys58, Thr85, Gln159, and Gly87 and displayed alkyl interactions with Pro307.

These results demonstrate that isoeleutherin, eleutherin, and eleutherol exhibit consistent and favorable binding to critical residues of EhOASS, some of which overlap with or complement those observed for metronidazole. This supports their potential role as modulators of this enzyme and strengthens the case of their amoebicidal activity.

### 3.2 Molecular Dynamics

The structural stability of the thioredoxin reductase enzyme (EhTrR) in its Apo form and in complex with the reference drug metronidazole as well as the compounds eleutherin, isoeleutherin, eleutherol, and isoeleutherol was investigated through 200 ns molecular dynamics (MD) simulations. The analyses included Root Mean Square Deviation (RMSD), Root Mean Square Fluctuation (RMSF), Radius of Gyration (Rg), and Solvent Accessible Surface Area (SASA), providing insights into the stability, flexibility, structural compactness, and solvent exposure of the protein–ligand systems.

The RMSD analysis revealed that Apo EhTrR exhibited greater structural fluctuation (4.61 ± 0.72 Å) compared to the ligand-bound complexes, indicating lower stability in the absence of a ligand. Metronidazole, the reference compound, showed a slightly lower RMSD value (4.43 ± 1.02 Å), suggesting moderate stabilization of the protein. Among the natural compounds, eleutherol induced the greatest structural stability, presenting the lowest RMSD (2.21 ± 0.47 Å), followed by isoeleutherin (2.24 ± 0.44 Å), eleutherin (2.58 ± 0.50 Å), and isoeleutherol (2.91 ± 0.60 Å) (Figure 4A).

**Figure 4:**
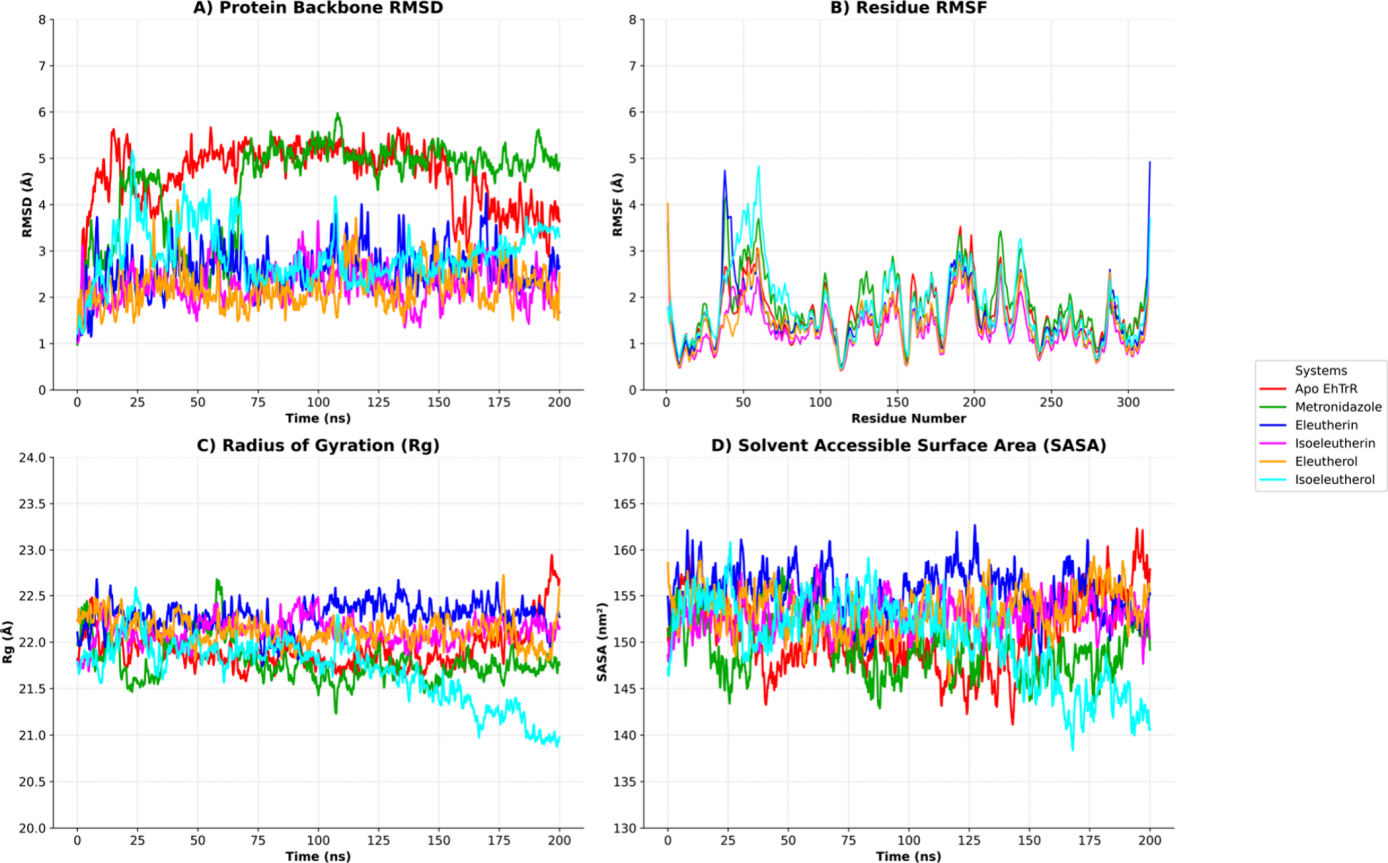
Molecular dynamics analysis of the thioredoxin reductase protein (EhTrR) in its Apo form and in complexes with the compounds Eleutherin, Isoeleutherin, Eleutherol, Isoeleutherol, and Metronidazole. (A) RMSD over 200 ns. (B) RMSF per residue. (C) Radius of Gyration (Rg). (D) Solvent Accessible Surface Area (SASA).

The RMSF results (Figure 4B) indicated that the Apo form of EhTrR had elevated residue-level fluctuations, particularly in the 40–65 region, which corresponds to loop segments and terminal regions. In contrast, the complexes with ligands exhibited attenuated fluctuations in these areas. Among them, isoeleutherol showed slightly higher fluctuations, which may reflect the local flexibility in its binding mode.

The Radius of Gyration (Rg) analysis demonstrated that the Apo protein underwent slight compaction during the simulation, with an average Rg of 21.92 ± 0.23 Å. The ligand-bound systems showed generally more expanded conformations, with average Rg values of 21.79 ± 0.23 Å for metronidazole, 22.28 ± 0.17 Å for eleutherin, 22.11 ± 0.16 Å for isoeleutherin, 22.12 ± 0.16 Å for eleutherol, and 21.69 ± 0.35 Å for isoeleutherol (Figure 4C).

Analysis of the Solvent Accessible Surface Area (SASA) showed that the Apo EhTrR had an average value of 15,083.7 ± 430.78 Å^2^, suggesting a more compact structure. The ligand-bound systems displayed slightly higher SASA values, reflecting an increased surface exposure upon ligand interaction. The complex with eleutherin exhibited the highest SASA (15,542.6 ± 306.27 Å^2^), followed by eleutherol (15,341.2 ± 296.98 Å^2^), isoeleutherin (15,283.7 ± 256.62 Å^2^), isoeleutherol (15,037.4 ± 484.44 Å^2^), and metronidazole (14,951.4 ± 358.21 Å^2^) (Figure 4D).

These findings suggest that natural compounds, particularly eleutherol and isoeleutherin, may confer structural stability to EhTrR, potentially enhancing its inhibition by restricting flexibility and promoting favorable conformational states.

Molecular dynamics simulations were performed to evaluate the structural behavior of O-acetyl-serine sulfhydrylase (EhOASS) in its Apo form and in complex with the natural compounds eleutherin, isoeleutherin, eleutherol, and isoeleutherol, as well as the reference drug metronidazole. The analyses focused on RMSD and RMSF to assess structural stability and flexibility, while Rg and SASA were used to examine conformational compactness and solvent accessibility, respectively.

The RMSD results indicated that the Apo EhOASS structure experienced the greatest backbone deviation during the simulation (3.19 ± 0.28 Å), suggesting higher structural instability in the absence of ligands. Upon ligand binding, reduced RMSD values were observed, with isoeleutherol and metronidazole presenting the lowest values (both 2.94 ± ∼ 0.3 Å), indicating enhanced stability. Conversely, the isoeleutherin complex showed the highest RMSD (3.77 ± 0.28 Å), followed by eleutherol (3.06 ± 0.23 Å) and eleutherin (2.98 ± 0.28 Å), as shown in Figure 5A.

**Figure 5:**
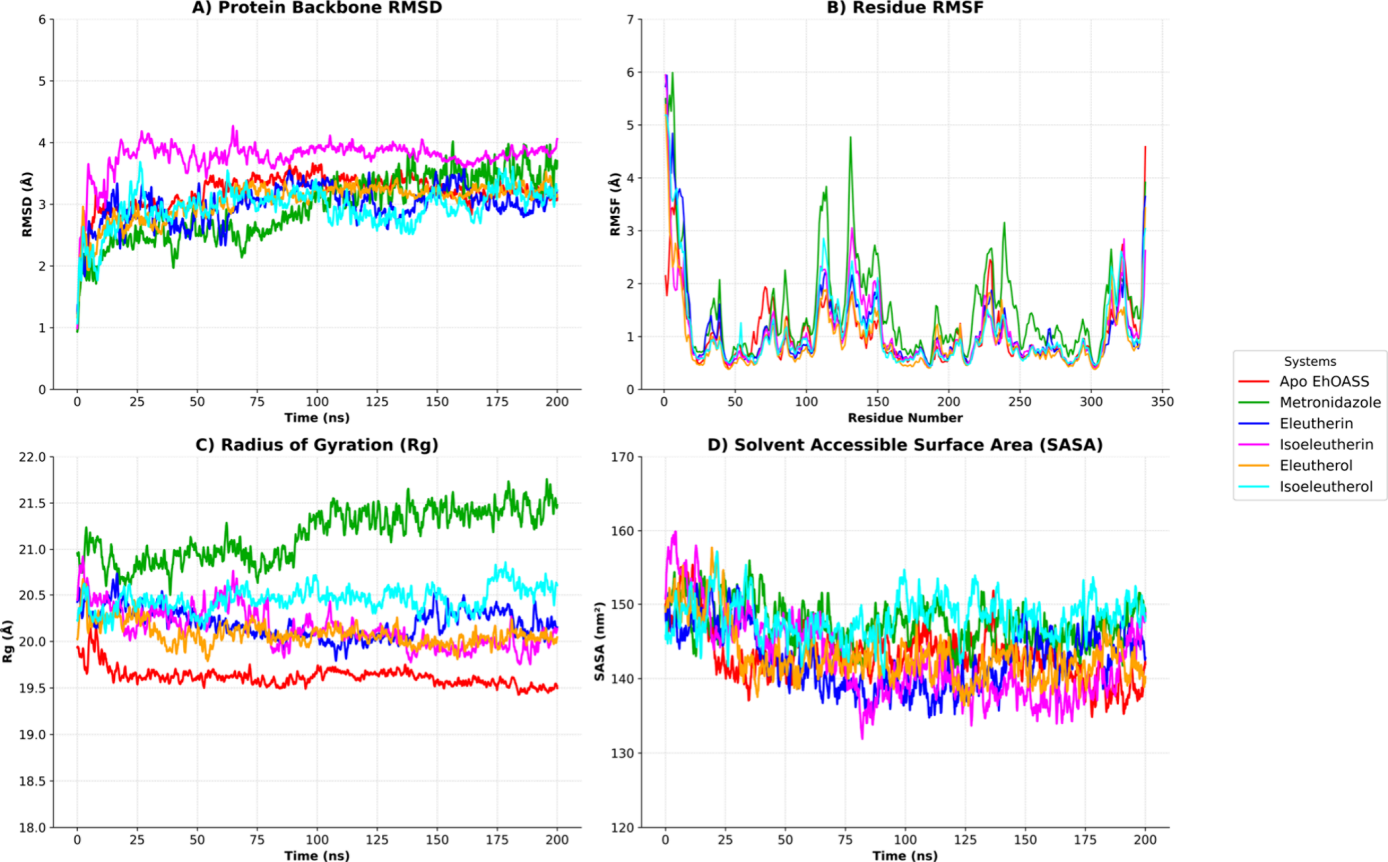
Molecular dynamics analysis of the complexes Eleutherin, Isoeleutherin, Eleutherol, Isoeleutherol, Metronidazole and the Apo protein for the second target enzyme, O-acetyl-serine sulfhydrylase (3BM5). (A) RMSD over 200 ns. (B) RMSF per residue. (C) Radius of Gyration (Rg). (D) Solvent Accessible Surface Area (SASA).

Residue-level flexibility assessed by RMSF (Figure 5B) revealed increased fluctuations in the Apo form, particularly in the terminal and loop regions. Among the complexes, eleutherol induced the lowest fluctuations across residues, indicating a stabilizing effect upon binding.

The Radius of Gyration (Rg) values provided further evidence of ligand-induced conformational changes. The Apo structure remained more compact (19.62 ± 0.11 Å), while ligand binding led to a slight increase in the molecular size. The highest Rg value was observed for the isoeleutherol complex (20.46 ± 0.14 Å), followed by metronidazole (21.16 ± 0.18 Å), eleutherin (20.20 ± 0.16 Å), isoeleutherin (20.15 ± 0.14 Å), and eleutherol (20.08 ± 0.16 Å) (Figure 5C).

SASA analysis (Figure 5D) revealed that most complexes exhibited a slight reduction in solvent exposure compared to the Apo form (143.17 ± 4.00 nm^2^), with the exception of isoeleutherol (148.19 ± 3.34 nm^2^) and metronidazole (147.41 ± 3.12 nm^2^), which showed increased solvent accessibility. These findings suggest that isoeleutherol may induce a more open and solvent-exposed protein conformation, potentially influencing the interaction dynamics.

Overall, the simulations suggest that ligand binding contributes to increased structural stability and alters the conformational behavior of EhOASS, with isoeleutherol and metronidazole standing out for their stabilizing effects and enhanced solvent exposure.

The binding affinity of eleutherin, isoeleutherin, eleutherol, and isoeleutherol compounds with the enzyme thioredoxin reductase (EhTrR) was evaluated using the MM/GBSA (Molecular Mechanics/Generalized Born Surface Area) method. The results showed significantly favorable free binding energy values (ΔGMM/GBSA), ranging from −23.47 (eleutherol) to −27.67 kcal/mol (isoeleutherin). In contrast, metronidazole showed a considerably lower affinity (ΔGMM/GBSA = −15.14 kcal/mol). These data reveal that all natural compounds evaluated show greater binding affinity for EhTrR compared to metronidazole, suggesting a more pronounced inhibitory potential.

The van der Waals energy component (ΔEvdW) was one of the main contributors to the stabilization of the complexes, being more favorable for eleutherin (−37.6464 kcal/mol) and isoeleutherin (−36.2358 kcal/mol). In contrast, eleutherol (−29.9266 kcal/mol) and isoeleutherol (−27.7979 kcal/mol) had smaller contributions. Metronidazole exhibited the lowest ΔEvdW value (−21.0314 kcal/mol), reinforcing its lower bond stability compared to natural compounds.

Electrostatic energy (ΔEele) varied significantly among the ligands, with isoeleutherol (−27.6202 kcal/mol) showing the most favorable contribution, followed by isoeleutherin (−7.082 kcal/mol) and eleutherin (−4.726 kcal/mol). Metronidazole, in turn, showed a minimal electrostatic contribution (−1.2565 kcal/mol), indicating that nonpolar interactions predominate in its binding.

Polar (ΔGGB) and nonpolar (ΔGnonpol) desolvation energies were also analyzed. Isoeleuterol stood out with the highest ΔGGB value (+30.6274 kcal/mol), while metronidazole showed a positive value for ΔGnonpol (+7.1490 kcal/mol), indicating a possible energy cost associated with its binding (Table 1)

**Table 1:** Binding Energy Components (kcal/mol) Calculated by the MM/GBSA. (EhTrR)

Molecules	Energetics Components (kcal/mol)				
	ΔEvdW	ΔEele	ΔG_GB_	ΔGnonpol	ΔGMM/GBSA
Metronidazole	–21.03 ± 0.15	–1.25 ± 0.35	7.15 ± 0.38	–22.28 ± 0.32	–15.14 ± 0.17
Eleutherin	–37.64 ± 0.08	–4.72 ± 0.11	15.14 ± 0.12	–42.37 ± 0.01	–27.22 ± 0.08
Isoeleutherin	–36.23 ± 0.09	–7.08 ± 0.09	15.64 ± 0.08	–43.31 ± 0.08	–27.67 ± 0.09
Eleutherol	–29.92 ± 0.06	–5.61 ± 0.09	12.07 ± 0.11	–35.54 ± 0.05	–23.46 ± 0.06
Isoeleutherol	–27.79 ± 0.08	–27.62 ± 0.17	30.62 ± 0.13	–55.41 ± 0.01	–24.79 ± 0.08

The evaluation of the binding affinity of the compounds with the enzyme O-acetyl-serine sulfhydrylase (EhOASS) revealed favorable interactions for all molecules tested, with free binding energies (ΔGMM/GBSA) ranging from −12.90 (eleutherin) to −20.30 kcal/mol (isoeleutherin). Isoeleutherin stood out as the compound with the highest affinity, followed by eleutherol (−19.41 kcal/mol), isoeleutherol (−17.83 kcal/mol), and eleutherin (Table 2). Comparatively, metronidazole presented an intermediate ΔGMM/GBSA value (−25.93 ± 0.23 kcal/mol).

**Table 2:** Binding Energy Components (kcal/mol) Calculated by MM/GBSA (3BM5)

Molecules	Energetics Components (kcal/mol)				
	ΔEvdW	ΔEele	ΔGGB	ΔGnonpol	ΔGMM-GBSA
Metronidazole	–25.73 ± 0.21	–28.51 ± 0.31	28.32 ± 0.26	–54.24 ± 0.31	–15.14 ± 0.17
Eleutherin	–25.55 ± 0.0862	–1.60 ± 0.11	17.58 ± 0.12	–3.32 ± 0.01	–12.90 ± 0.08
Eleutherol	–27.97 ± 0.06	6.52 ± 0.09	5.39 ± 0.09	–3.35 ± 0.01	–19.40 ± 0.06
Isoeleutherin	–27.32 ± 0.09	–3.71 ± 0.09	13.82 ± 0.08	–3.08 ± 0.01	–20.29 ± 0.09
Isoeleutherol	–23.61 ± 0.08	–7.63 ± 0.17	16.62 ± 0.13	–3.19 ± 0.01	–17.83 ± 0.08

Hydrophobic interactions, evaluated using van der Waals energy (ΔEvdW), were particularly pronounced for eleutherol (−27.97 ± 0.06 kcal/mol) and isoeleutherin (−27.32 ± 0.09 kcal/mol), while eleutherin and isoeleutherol showed slightly lower values (−25.55 and −23.62 kcal/mol, respectively). Metronidazole showed a comparable value (−25.73 ± 0.21 kcal/mol) in this energy component.

The analysis of the electrostatic energy (ΔEele) revealed marked differences between the compounds. While isoeleutherol showed a favorable contribution (−7.64 ± 0.17 kcal/mol), eleutherol showed a positive value (+6.53 ± 0.10 kcal/mol), indicating a possible electrostatic repulsion. Metronidazole stood out with the most favorable electrostatic contribution (−28.51 ± 0.32 kcal/mol).

The polar desolvation component (ΔGGB), generally unfavorable to binding, was more pronounced for eleutherin (17.58 ± 0.13 kcal/mol) and isoeleutherol (16.63 ± 0.13 kcal/mol). Nonpolar desolvation (ΔGnonpol), associated with hydrophobic effects, showed limited variation among the compounds (−3.09 to −3.36 kcal/mol), with metronidazole showing atypical behavior (ΔGnonpol = +7.15 ± 0.32 kcal/mol). (Table 2)

## 4 Discussion

Amoebiasis is a neglected tropical disease, with few studies aimed at the search for therapeutic alternatives. Factors such as resistance to current treatments, toxicity of available drugs, and the lack of effective options for some species highlight the need to explore new therapeutic strategies.^33^ Evaluating antiamoebic activity using in vitro or in vivo models is complex and faces several challenges, and there are no validated standard protocols for testing the activity of molecules against amoebae.^34^ In this context, molecular docking and dynamics can be valuable strategies for screening molecules to be submitted to in vitro and in vivo analyses against amoebae.

In the process of selecting molecules, the traditional use of *E. plicata* for the treatment of diarrhea associated with parasitic infections was considered,^11^ with eleutherol being the major compound and the other constituents being associated with various biological activities.^14,17^

To contextualize and validate this ethnobotanical use, a previous study demonstrated the in vitro antiamebic activity of the aqueous extract of *E. plicata* against strains of *Entamoeba histolytica* and *Entamoeba dispar* 42. In this study, complete inhibition of *E. histolytica* trophozoite growth was observed within 24 h in assays where metronidazole (2 μg/mL) was used as a positive control. Crucially, isoeleutherin was identified by LC-DAD as one of the main constituents of the extract and was associated with antiamoebic activity due to its known pro-oxidant properties typical of naphthoquinones.^41^

Another important aspect of this study was the selection of key targets that are essential for parasite survival. Amoebae do not efficiently absorb cysteine, which is crucial for protein synthesis and the maintenance of cellular homeostasis. Therefore, OASS (O-acetylserine sulfhydrylase) is essential for parasite survival, as it catalyzes the conversion of O-acetylserine and sulfide into l-cysteine^35^

Another important target for the selection of molecules with antiamoebic potential is EhTrR. *Entamoeba histolytica* faces a hostile environment in the human body, being exposed to ROS generated by the immune system, and EhTrR reduces oxidized proteins and protects the cell against oxidative damage.^36^ It is worth noting that isoleutherin has been shown to bind to trypanothione reductase (TrxR) present in trypanosomatids.^17^ Both enzymes (Trx and TrxR) are FAD- and NADPH-dependent and belong to the disulfide reductase superfamily^37^

Molecular docking analysis with the thioredoxin reductase (EhTrxR) protein revealed that isoeleutherin and eleutherin, in particular, have interaction profiles comparable to or even superior to those of metronidazole, interacting with residues located in the catalytic or functionally relevant region of the enzyme. The overlap in the hydrogen bond patterns relative to the reference compound further reinforces the potential of these molecules as candidate inhibitors. In addition, the interactions occur with conserved residues essential for the redox activity of the protein, suggesting possible interference with the biological function of EhTrxR.

The study also investigated a distinct target, O-acetyl-serine sulfhydrylase (OASS, PDB ID: 3BM5), a key enzyme in the biosynthetic pathway of l-cysteine. The results demonstrated that isoeleutherin, eleutherin, and eleutherol exhibit stable and favorable interactions with critical residues of EhOASS, some of which coincide with or complement those observed for metronidazole. These interactions involve residues essential for the enzyme’s catalytic activity, suggesting that the compounds may directly interfere with the metabolism of *E. histolytica*. The disruption of this metabolic pathway by natural compounds represents a promising strategy for the development of new therapies against amoebiasis.^38,42^

When evaluating which molecules were most promising for OASS, eleutherin and isoeleutherin showed more favorable profiles. The ligand–protein complexes exhibited reduced fluctuation patterns, suggesting restricted mobility due to compound binding. Isoeleutherol showed slightly higher residue fluctuations, which may indicate less favorable dynamic interactions and greater instability. The eleutherol complex presented slightly higher fluctuations in certain loop regions, which may be related to lower overall stability, as indicated by RMSD. However, the general trend of residue stabilization by isoeleutherol aligns with the results observed for the first target.

For EhTrR, the best interactions were observed with eleutherol and isoeleutherin. In this target, all complexes and the apo protein showed a similar fluctuation pattern, with notable peaks in the N-terminal and C-terminal regions. However, a slight reduction in fluctuations was observed for the complexes with isoeleutherol and eleutherin compared to the apo protein, indicating a stabilizing effect in these regions, especially in the central residues of the protein.

MM/GBSA energy calculations revealed distinct interaction profiles for the two enzyme targets. For EhTrxR, all natural compounds exhibited significantly more favorable binding energies (ΔGMM/GBSA between −23.47 and −27.67 kcal/mol) than metronidazole (−15.14 kcal/mol), with isoeleuterin emerging as the most promising ligand (−27.67 kcal/mol). This compound demonstrated particular efficiency in hydrophobic interactions (ΔEvdW = −36.24 kcal/mol) and a balanced electrostatic contribution (ΔEele = −7.08 kcal/mol).

For EhOASS, although metronidazole exhibited greater apparent affinity (−25.93 kcal/mol), its binding was marked by unfavorable nonpolar desolvation (ΔGnonpol = +7.15 kcal/mol), suggesting a significant energy cost. In contrast, Isoeleutherin maintained a competitive binding energy (−20.30 kcal/mol) with a more balanced energy profile, once again standing out among natural compounds. Particularly noteworthy was its combination of strong hydrophobic interaction (ΔEvdW = −27.32 kcal/mol) with a moderately favorable electrostatic contribution (ΔEele = −3.71 kcal/mol).

This consistent pattern positions Isoeleutherin as the most promising compound among the natural products evaluated for both targets, suggesting its potential as a core for the development of multitarget inhibitors. Its ability to maintain high binding affinity through complementary mechanisms (strong hydrophobic interactions with EhTrxR and energy balance with EhOASS) may represent a strategic advantage in combating *E. histolytica*, simultaneously targeting essential metabolic pathways.

Considering both targets, Isoeleutherin appears to be the main molecule involved in antiamoebic activity and should be prioritized in vitro and in vivo studies. When comparing the toxicity of eleutherin and Isoeleutherin, eleutherin appears to be more cytotoxic and genotoxic in in vitro models 12, while also being the least active. On the other hand, the toxicities of isoeleutherol and eleutherol remain poorly explored, and further studies are necessary to validate their safety. Interestingly, eleutherol showed greater in vivo activity against *Plasmodium berghei* than in vitro,^16^ suggesting that it may act as a prodrug. Therefore, it is essential to assess the toxicity of eleutherol and its metabolites, including the use of the S9 fraction in in vitro toxicity studies.

In this context, our findings on isoeleutherin not only showed it to be the most promising compound in silico in terms of binding affinity to the EhTrR and EhOASS targets but also were previously characterized as having a lower toxicity profile, being even less cytotoxic and genotoxic than eleutherin itself in in vitro models. This combination of high potential activity and lower toxicity suggests that isoeleutherin may offer a significant therapeutic advantage over currently available drugs, paving the way for the development of safer and more effective treatments for amoebiasis. Additionally, this perspective is reinforced when considering the challenges of metronidazole therapy, which include the emergence of resistance and a wide spectrum of adverse effects such as gastrointestinal, psychiatric, neurological, and visual disorders.^10^

Relating the results of this study to the traditional use of *E. plicata* for the treatment of amoebiasis, it is possible to suggest that its activity may be linked to the synergistic effect of isoeleutherin, eleutherin, and eleutherol. The use of active molecule combinations can be an important strategy for preventing parasite resistance,^39^ as well as enhancing pharmacological effects.^40^ However, it is important to emphasize the need for in vitro and in vivo studies to evaluate whether synergy between the molecules also occurs in terms of toxicity.

## 5 Conclusions

Based on the results of molecular docking, molecular dynamics, and free energy, it is suggested that the most promising compound in terms of amoebicidal potential was the Isoeleutherin molecule targeting EhTrR and EhOASS. In addition, it has a lower toxicity profile. We emphasize that the search for therapeutic alternatives for the treatment of amoebiasis is of great importance, yet few projects have been developed for this purpose. The first challenge lies in cultivating the parasite to perform preliminary in vitro assays. Additionally, amoebiasis predominantly affects populations in situations of social vulnerability and with limited access to medicines, making research funding for this purpose economically unfeasible. Given these factors, a promising strategy in the search for amoebicidal molecules is the use of in silico studies in which only molecules with suitable pharmacokinetic and toxicological profiles, as well as the ability to act on specific parasite targets, are selected. This screening is then followed by in vitro and in vivo validation.

## Abbreviations

APBS: Adaptive Poisson–Boltzmann Solver
CAT: Catalase (enzima antioxidante)
DFT: Density functional theory
EhOASS: Entamoeba histolytica O-acetylserine sulfhydrylase
EhTrR: *Entamoeba histolytica* thioredoxin reductase
FAD: Flavin adenine dinucleotide
GAFF: General Amber Force Field
GPx1: Glutathione peroxidase 1
GR: Glutathione reductase
MD: Molecular dynamics
MM/GBSA: Molecular mechanics/generalized born surface area
MVD: Molegro Virtual Docker
NADPH: Nicotinamide adenine dinucleotide phosphate
NOS: Nitric oxide synthase
PDB: Protein Data Bank
PME: Particle Mesh Ewald
RESP: Restrained electrostatic potential
RMSD: Root mean square deviation
RMSF: Root mean square fluctuation
ROS: Reactive oxygen species
SASA: Solvent accessible surface area
